# Macular Optical Coherence Tomography Imaging in Glaucoma

**DOI:** 10.18502/jovr.v16i3.9442

**Published:** 2021-07-29

**Authors:** Alireza Kamalipour, Sasan Moghimi

**Affiliations:** ^1^Hamilton Glaucoma Center, Shiley Eye Institute, Viterbi Family Department of Ophthalmology, University of California, San Diego, La Jolla, CA, United States

**Keywords:** Artificial Intelligence, Glaucoma, Imaging, Macula, Optical Coherence Tomography

## Abstract

The advent of spectral-domain optical coherence tomography has played a transformative role in posterior segment imaging of the eye. Traditionally, images of the optic nerve head and the peripapillary area have been used to evaluate the structural changes associated with glaucoma. Recently, there is growing evidence in the literature supporting the use of macular spectral-domain optical coherence tomography as a complementary tool for clinical evaluation and research purposes in glaucoma. Containing more than 50% of retinal ganglion cells in a multilayered pattern, macula is shown to be affected even at the earliest stages of glaucomatous structural damage. Risk assessment for glaucoma progression, earlier detection of glaucomatous structural damage, monitoring of glaucoma especially in advanced cases, and glaucoma evaluation in certain ocular conditions including eyes with high myopia, positive history of disc hemorrhage, and certain optic disc phenotypes are specific domains where macular imaging yields complementary information compared to optic nerve head and peripapillary evaluation using optical coherence tomography. Moreover, the development of artificial intelligence models in data analysis has enabled a tremendous opportunity to create an integrated representation of structural and functional alterations observed in glaucoma. In this study, we aimed at providing a brief review of the main clinical applications and future potential utility of macular spectral-domain optical coherence tomography in glaucoma.

##  INTRODUCTION

Glaucoma, characterized by progressive loss of retinal ganglion cells (RGCs) and their axons accompanied by concomitant characteristic visual field (VF) damage, is globally a leading cause of irreversible blindness.^[1,2,3,4,5]^ Earlier diagnosis and monitoring of disease progression are two fundamental tasks for clinicians managing glaucoma.^[2,6,7]^ Developments in imaging modalities in the last three decades have led to recent advances in glaucoma diagnosis and management.^[8,9,10,11,12,13,14,15]^ Optical coherence tomography (OCT) is now the imaging modality of choice for objective assessment of glaucomatous structural alterations due to fast and highly reproducible scan acquisition.^[16,17,18]^ In clinical practice, most attentions on OCT imaging in glaucoma has been paid to the evaluation of the optic disc that together with VF assessment using 6° apart test points (e.g., the 24–2 or 30–2 test pattern) constitute the common clinical paradigm.^[19,20]^ However, evidence shows that sole reliance on common clinical paradigm might be insufficient in certain clinical aspects. Importantly, glaucomatous damage to the macular area may happen early in the disease course and such damage can be underestimated or even missed using common clinical paradigm.^[19]^ Also, evaluating glaucoma progression especially at the advanced stage where optic nerve head (ONH) and circumpapillary retinal nerve fiber layer (cpRNFL) measurements have reached to an apparent floor is limited by the current common clinical paradigm. Another challenge happens in the evaluation of glaucoma patients with high myopia where the OCT segmentation algorithms are more prone to errors at the ONH area as a result of anatomical alterations such as peripapillary atrophy, ONH tilt, and stretching of the cpRNFL. Macular OCT imaging with high resolution scans of different layers can be a useful adjunct to common clinical paradigm in these scenarios. Moreover, the application of artificial intelligence (AI) techniques to analyze the big data obtained from these high-resolution images shows promise to improve the currently available diagnostic modalities and structure–function relationships in glaucoma.

Glaucomatous damage to the macular area that contains around 50% of retinal RGCs in a multilayered fashion has been reported for a long time using histological studies.^[18]^ Potential damage to this area leads to impairments in the central VF which is associated with a dramatic decline on the functional status in glaucoma patients. Moreover, macular evaluation in glaucoma has recently regained a specific focus of interest based on the possibility of early involvement in the disease process.^[21]^ New SD-OCT post acquisitional algorithms provide automated segmentation of different layers of macular area that focuses on interest in glaucoma evaluation and monitoring including macular retinal nerve fiber layer (mRNFL), ganglion cell layer (GCL), ganglion cell/inner plexiform layer (GC/IPL), and ganglion cell complex (GCC). Consequently, the utility of these SD-OCT-derived macular parameters for glaucoma detection and monitoring of disease progression have been shown in many previous studies. In this perspective, we aim at providing more insight on the potential utility of SD-OCT macular imaging in glaucoma practice.

### Different Macular OCT Imaging Instruments

#### Cirrus high-definition OCT (HD-OCT)

**Figure 1:**
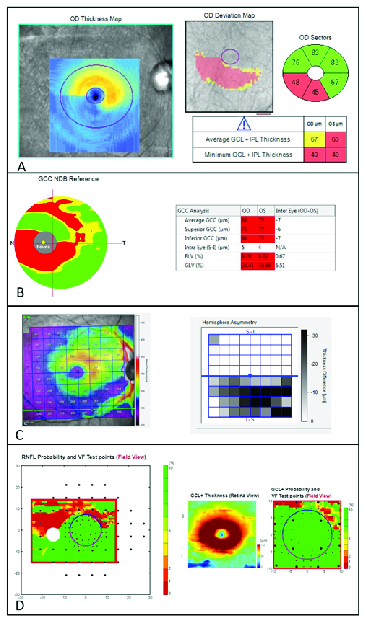
(A) Cirrus HD-OCT Ganglion Cell OU analysis of a patient with glaucoma. Thickness Map, Deviation, Sector Map, and average/minimum GCIPL can be provided by Cirrus OCT. Various types of optic neuropathy, including compressive optic neuropathy and ischemic optic neuropathy, can affect the macula and ganglion cell inner plexiform layer (GCIPL). However, in glaucoma, the inferotemporal region is frequently affected first. The temporal raphe sign is an important sign for distinguishing glaucoma from other neuropathies. The temporal raphe sign is positive if there is a horizontal straight line longer than one-half of the inner-to-outer-annulus length on the macular GCIPL thickness map. (B) Optovue ONH/GCC OU report of a glaucoma patient. Ganglion cell complex (GCC) significant map shows thinning of inner macula layers in the inferior regions. Tabular data also provides the average GCC, as well as focal loss volume (FLV) and general loss volume (GLV). (C) Heidelberg Posterior Pole Asymmetry Analysis Report: Thickness of whole retina as well as individual layers will be shown on an 8 × 8 grid and Hemisphere Asymmetry Map. Thinning of inferotemporal macula is evident in both maps. (D) Topcon Wide Glaucoma report provides GCIPL data (GCL+) or GCC data (GCL++, not shown) in the macula and optic nerve and can be combined with VF results (Hood Glaucoma report).

Cirrus HD-OCT (Carl Zeiss Meditec, Dublin, CA) is one of the instruments that provides high macular images using SD-OCT technology [Figure 1A]. The Cirrus HD-OCT performs volumetric scan (200 × 200 or 512 × 128 A-scans) of the macula over an area of 6 × 6 × 2-mm ${}^{3}$ in an emmetropic eye that is centered on the fovea. In the Ganglion Cell Analysis (GCA) printout, it provides the GCIPL parameter that includes the combined thickness measurements of GCL and IPL. GCA printout displays global average GCIPL, minimum GCIPL, and sectoral GCIPL measurements that are presented over six wedge-shaped regions bound by a horizontally oval area (4.8 × 4.0 mm ${}^{2}$ ) after the removal of a central perifoveal ellipse (1.2 × 1.0 mm ${}^{2}$ ). In addition to the mentioned parameters, GCA provides a color-coded deviation map of GCIPL measurements over the aforementioned elliptical area that compares localized thickness measurements to age-adjusted normative database of the built-in software.^[18]^

**Figure 2:**
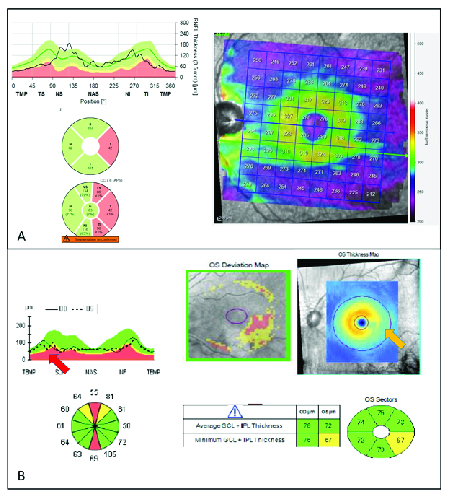
(A) Myopic eye without glaucoma. Myopic eye shows “red disease” with abnormal sectors in the inferotemporal, supratemporal, and temporal sectors which is probably due to temporal displacement of RNFL peaks and not glaucomatous damage. This is confirmed by normal macula thickness in the Posterior Pole Map. (B) Myopic eye with glaucoma. The OCT image shows thinning of the inferior and superior quadrants. This “red disease” might be due to the temporal displacement of RNFL peak. However, GCIPL report shows typical raphe sign (yellow arrow) and inferotemporal macula inner layer thinning suggesting glaucomatous damage. Detailed examination of RNFL profile also depicts a decrease in thickness of superior peak (red arrow).

**Figure 3:**
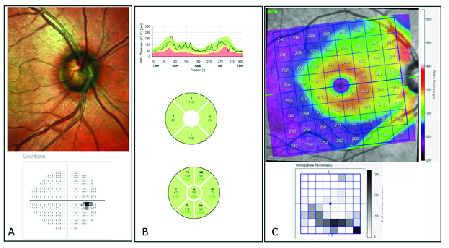
An early glaucoma case with normal VF (A) in which Spectralis RNFL Single report shows normal mean RNFL thickness values and normal sectoral value (B). Posterior Pole Thickness Map and Hemisphere Asymmetry Map shows area of thinning in the macular area corresponded to the location of narrow RNFL defect in optic disc photo.

#### RTVue SD-OCT

The RTVue (Optovue Inc., Fremont, CA) is another SD-OCT imaging modality that is capable of performing a 3-D volumetric scan of the macula over a 7mm square that is centered 0.75 mm temporally to the fovea. In the printout, it displays average, superior, and inferior GCC (including the combination of mRNFL, GCL, and IPL) measurements [Figure 1B]. Moreover, two color-coded maps are provided in the software output that display the absolute GCC measurements and GCC deviation patterns based on the age-adjusted normalized database of the software. Cross-sectional high-resolution B-scans are also shown for further structural evaluation and detection of possible image artifacts.^[18]^

#### Spectralis SD-OCT

Spectralis OCT (Heidelberg Engineering GmbH) instrument uses Posterior Pole Analysis (PPA, or Posterior Pole Asymmetry Analysis) algorithm to capture macular images composed of 61 distinct horizontal B-scans (X768 A-scans) that are aligned in parallel to Bruch's membrane opening (BMO)-fovea axis. Each horizontal B-scan is repeated 9–11 times and averaged to decrease speckle noise. The latest software (Glaucoma Module Premium Edition) provides automated segmentation of the layers of interest. The output displays an 8 × 8 thickness grid (64 superpixels, 3° wide) for each layer of interest and enables direct comparison between the superpixels of the fellow eyes as well as the corresponding superior and inferior superpixels of the same eye. Currently, no comparison to the normative data is available on the PPA^[18]^ [Figure 1C].

#### Topcon 3D-OCT

There are multiple Topcon 3D-OCT (Topcon, Inc., Paramus, NJ) instruments of different generations including 3D-OCT 1000, 3D-OCT 2000, and a newer swept-source OCT (DRI OCT-1) device. Topcon 3D-OCT measures a 6 × 6 mm ${}^{2}$ area with a protocol of 128 × 512 A-scans/image. DR OCT-1 performs faster measurements and is capable of acquiring wide-field scans over a 12 × 9 mm ${}^{2}$ area with 256 × 512 A-scans/image protocol. In the wide-field report, measurements of mRNFL, GCIPL, and GCC are presented^[18]^ [Figure 1D].

### Utility in the Detection of Early Glaucoma

It is well-known that disease severity affects the diagnostic performance of OCT parameters in glaucoma^[22,23,24,25]^ which is mostly represented using the Area Under the Receiver Operating Characteristic curve (AUROC). Hence, attempts have been made to evaluate the diagnostic accuracy of macular OCT parameters and compare their performance to those of ONH and cpRNFL thickness at earlier (pre-perimetric and mild perimetric) stages of glaucoma. Their findings reveal excellent and comparable diagnostic performance of macular and cpRNFL parameters in early glaucoma.^[26,27,28,29,30,31,32,33,34]^ In one of these studies, Kim et al found inferotemporal GCIPL as the macular parameter with the highest diagnostic performance (AUROC = 0.82) comparable to those of best parameters of RNFL (7 o'clock sector, AUROC = 0.76) and ONH (rim area, AUROC = 0.77).^[32]^ Other studies have reported comparable diagnostic performances of macular GCIPL and cpRNFL deviation maps in the detection of pre-perimetric glaucoma and good performance of macular GCA maps in the detection of early glaucoma (VF mean deviation [MD] > –6dB) with the detection rate of up to 87.8% (in the deviation map).^[28,33]^ Importantly, it is shown that glaucomatous damage to the macular region can happen early in the disease course and sole reliance on a combination of 24-2 VF tests and optic disc OCT can underestimate or even miss the damage.^[19]^ With this respect, Kim and colleagues studied a group of 186 glaucoma patients using Cirrus HD-OCT instrument and found that in a subgroup of patients, defects are evident on macular GCIPL but not on cpRNFL deviation maps. However, all cases with a defect on cpRNFL deviation map had a corresponding defect on macular GCIPL deviation map. Based on these findings, they suggested that macular OCT is capable of identifying early glaucomatous damages that may not be apparent on OCT scans of the disc area.^[35]^

Another useful sign for the diagnosis of early glaucoma in macular OCT is the presence of temporal horizontal raphe on GCIPL deviation maps [Figure 1A]. It is defined based on the intuition that early glaucomatous damage preferentially affects one hemifield more than the other. Kim et al developed GCIPL hemifield test which is an automated program for the detection of glaucoma based on this finding. They showed that this test has a very high diagnostic performance for the detection of pre-perimetric glaucoma (AUROC = 0.97) and early perimetric glaucoma (AUROC = 0.96).^[36]^ Moreover, in a separate study it was shown that the presence of this sign can be a useful indicator to discriminate glaucomatous from other non-glaucomatous causes of optic neuropathy in eyes with GCIPL thinning.^[37]^ However, these findings from separate groups of Asian glaucoma patients with a presumable high prevalence of normal tension glaucoma may not be generalizable to glaucoma patients of other ethnicities.

The association between distinct glaucomatous optic disc appearance and the presence of central VF defects has been a recent focus of interest. Ekici and collaborators demonstrated that early macular damage in glaucoma patients tends to happen more in optic discs with focal ischemic and myopic phenotypes compared to those with generalized cup enlargement phenotype.^[38]^ Likewise, eyes with optic disc hemorrhage have been associated with more degree of central macular involvement and parafoveal scotoma. In a study by Liu et al, GCIPL showed higher proportional rates of thinning and greater association with functional progression compared to cpRNFL.^[39]^ Accordingly, macular structural evaluation using OCT can be tailored to the individual patients based on the optic disc appearance and this has the potential to enhance the diagnosis and management of early glaucoma.

### Monitoring of Advanced Glaucoma

Monitoring of advanced glaucoma is another challenging task in clinical practice where VF tests show increased variability. Evaluating structural change alongside functional performance can be a useful adjunct if the measurements are reproducible, demonstrate acceptable global and regional structure–function relationship, and fall within the dynamic range of variability. The concept of measurement floor points to another limitation in the evaluation of advanced glaucoma. It is ascribed to a level beyond which the structural measurements no longer show a decremental pattern with worsening of the disease and is believed to represent the thickness of the remining non-neural tissues. Despite that the dynamic range for cpRNFL extends to –8 to –10 dB MD, macular OCT reaches measurement floor at more advanced glaucomatous damage.^[18,40]^ Bowd and collaborators defined a region of interest approach to estimate the measurement floors of optic disc and macular parameters in a longitudinal cohort of moderate to advanced glaucoma patients (MD ≤ –8 dB). They found that the baseline region of interest percentage above the measurement floor for advanced glaucoma cases (MD ≤ –12 dB) was highest for GCIPL thickness (36%), followed by minimum rim width (19%) and cpRNFL thickness (14%). As a conclusion, they suggested that macular GCIPL thickness might be a better candidate for monitoring progression in advanced glaucoma compared to optic disc parameters.^[41]^ In another study, Belghith and colleagues found that only GCIPL thickness (compared to cpRNFL) had a significantly faster rate of progression in highly advanced glaucoma patients (MD < –21 dB) compared to healthy subjects.^[42]^ Similarly, Lavinsky et al based on an average of four years of follow-up including eyes with a median MD of –10.2 dB reported an average –0.57 µm/year decline in GCIPL thickness compared to a nonsignificant rate of change for cpRNFL (0.009 µm/year).^[43]^

In addition, macular OCT measurements have shown high reproducibility in advanced disease, good correlation with VF sensitivity measures, and preserved dynamic range at a stage when the reliability of VF tests decline. A small number of studies have shown that the variability of macular measurements does not significantly increase as the disease gets worse.^[44,45]^ This recommends macular measurements like GCC and GCIPL thickness as possible biomarkers for the evaluation of advanced glaucoma; however, it must be kept in mind that accurate segmentation of different macular layers becomes more challenging with worsening of the disease.^[18]^

### Utility in the Detection of Glaucoma in Myopic Eyes

Evaluation of glaucoma in myopic eyes is another area where macular images may yield additional benefit compared to ONH images. Evaluation and diagnosis of glaucoma in highly myopic eyes is challenging. It has been shown that ONH parameters have a worse performance in the detection of glaucoma in highly myopic eyes compared to non-highly myopic eyes while GCC's performance remains the same.^[46]^ We know that myopia affects the patterns of RNFL distribution measured by the SD-OCT instruments resulting in temporal displacement of RNFL peaks on thickness plots with possible influence on the diagnostic performance of OCT measurements.^[47]^ Therefore, the so-called “red disease” is not uncommon when clinicians evaluate ONH repots in these patients [Figure 2A]. This false positive in cpRNFL color code is especially common in inferior quadrants.^[48]^ In addition, the morphology of the ONH might be altered in highly myopic patients as a result of ONH tilt and the presence of peripapillary atrophy. These anatomical changes may lead to OCT artifacts^[49]^ and potentially affect the performance of segmentation algorithms especially in the ONH due to a more complex anatomy compared to the macula.^[50,51]^ In the same line, investigators have shown the superiority of macular over ONH parameters for the diagnosis of glaucoma in highly myopic eyes;^[52,53,54,55,56]^ although, there are some reports showing a comparable performance between the measurements of these two areas.^[29,57,58,59,60]^ Inferotemporal GCIPL thickness is the best macular parameter to detect glaucomatous damage in highly myopic eyes especially at the pre-perimetric stage ^[57]^ [Figure 2B]. Kim and colleagues studied Asian high myopic patients and demonstrated an excellent diagnostic accuracy for GCIPL hemifield test to detect glaucomatous damage (AUROC = 0.94).^[59]^ Hence, the presence of temporal horizontal raphe on GCIPL thickness map in high myopic eyes may serve as a useful diagnostic clue to detect glaucoma.

### Risk Assessment

As glaucomatous VF damage is generally irreversible, early intervention is required to prevent further functional deterioration and potential blindness.^[61,62]^ This highlights the role of earlier disease detection and risk stratification of patients according to the future rates of glaucoma progression. Clinical parameters that are associated with prognostic utility in terms of future glaucoma progression are age, the level and fluctuation of intraocular pressure, central corneal thickness, disc hemorrhage, and the diagnosis of pseudoexfoliative glaucoma.^[63,64,65,66,67,68,69,70,71]^ Certain high risk groups may not only benefit from earlier intervention, but also require closer follow-up appointments and diagnostic tests for the evaluation of disease progression. Macular OCT imaging can be a useful modality in some of these patients. Recently, Shukla and colleagues^[72]^ showed that the presence of disc hemorrhage is associated with more severe damage on 10-2 VF test and faster rate of central VF progression. Hence, this subgroup of patients may benefit from regular macular OCT monitoring for earlier detection of glaucoma progression. Another study showed that the presence of temporal raphe sign on baseline macular GCIPL deviation maps of elderly patients with enlarged vertical cup-to-disc ratio is associated with faster progression to normal tension glaucoma.^[73]^ In addition to the aforementioned clinical settings, it has been shown that evaluating macular structure using OCT can further enhance the performance of prognostic models and consequently improve our understanding of glaucoma progression. Anraku and associates evaluated the performance of different baseline structural (macular and cpRNFL OCT) and functional parameters in a cohort of early glaucoma eyes for the detection of future VF progression. The cohort was classified into two groups of slow (MD rate > –0.4 dB/year) and fast (MD rate < –0.4 dB/year) progressors. Only thinner macular GCC at baseline was a significant predictor of future fast progression.^[74]^ Moreover, in two separate studies, Zhang and colleagues demonstrated that among baseline SD-OCT measurements, GCC focal loss volume (FLV) is the best single predictor for subsequent glaucoma conversion in pre-perimetric glaucoma patients^[75]^ and for VF progression in patients with established glaucoma.^[76]^ In the first study, they found that eyes with abnormal or borderline GCC–FLV have a four-fold increase in the risk of future glaucoma conversion over a six-year period. In the second study, they reported that abnormal GCC–FLV at baseline leads to a triple increase in the risk of future VF progression based on an average 3.7 years follow-up of 277 eyes with established glaucoma and average baseline VF MD of –4.8 dB. Hou et al evaluated the temporal relationship between progressive GCIPL thinning, cpRNFL thinning, and VF progression in a cohort of patients with primary open-angle glaucoma with a follow-up duration of more than five years. They found that progressive GCIPL and cpRNFL thinning are mutually predictive and both are indicative of VF progression.^[77]^ They suggested that integrating macular and cpRNFL parameters may probably lead to earlier detection of disease progression in glaucoma patients.

### Applications of Artificial Intelligence (AI)

In the recent years, the applications of AI in general (and deep learning networks in particular) into medicine has led to the introduction of numerous automated diagnostic modalities. AI techniques have many implications in machine vision tasks including image classification with the performance sometimes higher than that of humans^[78]^ and unsupervised identification of different patterns that exist in large datasets of images. A widespread use of different imaging modalities in ophthalmology research and clinical practice makes this medical subspecialty a major area for the implementation of these novel algorithms to assist in diagnosis and improve the currently used image analysis techniques.^[79]^ A great proportion of publications on AI methods in glaucoma have focused on the detection of the disease using different inputs like fundus and OCT images. Asaoka and colleagues developed and validated a deep learning model to accurately (AUROC = 93.7%) detect early open-angle glaucoma (MD > –5 dB) using macular OCT information.^[80]^ Another recent study developed a 3D deep learning system to detect patients that need to be referred to a glaucoma specialist based on the volumetric macular OCT information. The overall accuracy of their proposed surveillance system was high (AUROC = 0.88) with a relatively well preserved performance among eyes with different degrees of myopia.^[81]^ Development of deep learning models to evaluate the details of structure–function relationship has been another focus of AI investigations in glaucoma. These models have shown a high accuracy to extract and use the relevant information obtained from macular volumetric OCT scans and provide a corresponding simulation of central VF in glaucoma patients.^[82,83,84]^ Moreover, Nouri-Mahdavi et al in a recent study showed that VF progression in moderate to advance glaucoma can be partly predicted using combined OCT measurements of peripapillary and macular areas. Of note, they developed and compared separate models using macular or peripapillary measurements and showed that macular models performed better than peripapillary models to detect VF progression. This finding highlights the potential of macular OCT in monitoring patients with moderate to advanced glaucoma.^[85]^ Hopefully, by further refining these AI approaches, automated precise systems for the detection and monitoring of disease progression in glaucoma will become available in the future.

### What We Should Do as Clinicians

While it is common clinical practice to obtain an OCT scan of the disc, many clinicians do not routinely obtain a scan of the macular region for patients with glaucoma or suspected glaucoma. As previously discussed, macula SD-OCT has been helpful for the earlier detection of glaucoma – particularly in eyes with certain ONH phenotypes, eyes with DH, and myopic eyes. For example, Figure 3 shows a case in which the cpRNFL report appears normal, while the macula scan shows apparent GCIPL thinning. Macula scans may also serve as a clue for clinicians for the presence of parafoveal scotoma, which should receive attention and be further evaluated with a 10-2 VF test. In addition, macula scans play an important role in monitoring eyes with advanced disease, as they may help clinicians identify disease progression and decide to escalate treatment. Consequently, one may ask, “When do you perform an OCT scan of the macula?” Although macula scans can be selectively ordered for those patients who may be most likely to benefit from it (as discussed earlier), obtaining OCT scans is so efficient today that many experts recommend routinely performing both disc and macula scans for all patients with glaucoma and suspected glaucoma, thus have a comprehensive glaucoma assessment of the patients.

##  Limitations

Like every other imaging modality, there are some limitations in the use macular OCT for glaucoma practice and research that clinicians need to consider. First, GCIPL analysis with the SD-OCT may be complicated by coexisting macular pathology and scan artifacts. Most currently available data on macular OCT in glaucoma are obtained from studies that have excluded eyes with other macular pathologies and also poor quality images like those with artifacts and lower signal strength. Thus, one needs to expect higher variability of macular measurements in real world scenarios. The presence of age-related macular pathologies and drusen may disrupt the correct segmentation of the macular layers that are important in the diagnosis and monitoring of glaucoma patients especially in the elderly. Recent studies using GCIPL analysis have excluded up to 6% of scans due to machine segmentation or acquisition error.^[86]^ Moreover, any retinal diseases involving macular areas such as the epiretinal membrane, age-related macular degeneration, or macular edema can affect macular GCIPL thickness and reducing performance of the macula scans for detection of glaucoma. Similarly, macula scans are not helpful for detection of glaucoma in eyes with myopic myopathy. Even in eyes without maculopathy, abnormal diagnostic classifications on GCIPL map can be seen in up to 40% of myopic eyes with diffuse, circular pattern being the predominant form.^[87]^ In addition, macular measurements obtained by different OCT devices are not interchangeable despite showing a fair degree of correlation.^[88]^ Finally, it has to be mentioned that glaucomatous damage with a high angular distance from the BMO–fovea axis may fall out of the measurement territory of macular OCT images depending on the OCT instrument and software and consequently not be identifiable on the deviation maps. Clinicians need to be aware of this issue and take it into consideration.^[28,32]^

##  CONCLUSION

Macular OCT is a useful imaging modality in glaucoma management and research. It provides complementary information to the conventionally used modalities by glaucoma specialists especially in the evaluation of patients with early macular damage and/or high myopia and monitoring of the advanced disease. With the development and widespread use of AI techniques in medicine, macular OCT information can be integrated with the information obtained from optic disc OCT and VF assessment to provide a more comprehensive picture of the true nature of glaucomatous damage and progression. This will definitely enhance the quality of medical care and research in the future.

##  Financial Support and Sponsorship

This study was supported by the UC Tobacco Related Disease Research Program Grant T31IP1511.

##  Conflicts of Interest

There are no conflicts of interest.
